# Prolonged time to breast cancer surgery and the risk of metastasis: an explorative simulation analysis using epidemiological data from Germany and the USA

**DOI:** 10.1007/s10549-025-07630-9

**Published:** 2025-02-17

**Authors:** D. Hölzel, A. Schlesinger-Raab, G. Schubert-Fritschle, K. Halfter

**Affiliations:** Faculty of Medicine, Institute of Medical Information Processing, Biometry and Epidemiology (IBE), LMU Munich, Marchioninistraße 15, 81377 Munich, Germany

**Keywords:** Breast cancer, Surgery, Treatment delay, Metastasis, Long-term follow-up

## Abstract

**Purpose:**

Growing breast cancer is associated with an inherent risk of metastasis. If surgical treatment of breast cancer is delayed, the prognosis worsens with increasing tumor size. This justifies the search for a safe time interval between diagnosis and surgery.

**Methods:**

The 2022 population-based data on incidence and the time interval to initial surgery for the United States (U.S.) and Germany are used. Tumor growth and initiation of metastases can be calculated using public data on hormone receptor status, volume doubling time, and tumor size-dependent relative survival. Our assumptions are based on an initial 19.8 mm mean tumor size. 15-year BC-specific mortality in both countries is assumed to be 19.6% without surgical delay. Volume doubling time stratified by hormone receptor status, assumed to be continuous may differ by a factor of 2.4.

**Results:**

The U.S. and Germany report 287,850/71,375 new breast cancers for the year 2022 and 2019. If tumor removal is delayed by 8 weeks, mortality rate increases by 2.25/4.79% (HR + /HR−) as estimated by our model. The currently reported mean delay in the U.S. and Germany of 33.7/26.0 days or 4.8/3.7 weeks, respectively, would lead to an estimated 4,676/918 additional BC deaths or a 1.6/1.2% rise in the 15-year BC-specific mortality rate.

**Conclusions:**

This study offers reasonable evidence that confirmed cases of breast cancer should be prioritized and treated according to hormone receptor status and tumor size as soon as possible. Effective screening measures should be followed by timely treatment.

**Supplementary Information:**

The online version contains supplementary material available at 10.1007/s10549-025-07630-9.

## Introduction

The question of how the risk for breast cancer patients increases with the prolongation of the time between diagnosis and surgery has been examined many times, most recently because of interruptions in health care during the COVID-19 pandemic [1]. Based on the lessons learned from the pandemic, a similar systematic disruption of the health care system in the future is unlikely. Yet, looking at data from the United States (U.S.) and Germany, the time between breast cancer (BC) diagnosis and primary treatment continues to increase. [2] Currently, an eight-week time period between initial diagnosis and surgical intervention is stated as an upper safe time limit in BC [3]. This treatment indicator contradicts studies which demonstrate worsening prognosis with delayed treatment. For example, in a systematic review and meta-analysis Hanna et al. found that a four-week cancer treatment delay across all common treatment modalities, including breast cancer, is associated with an increase in mortality in seven different cancer entities.[4, 5]. The 15-year mortality in BC patients was increased by 8% (95%CI 3–13%) for each four-week delay. Considering the already extended time between first suspicion, initial diagnosis, and treatment in the last decade, the effect of a delay in treatment may even be underestimated in 2024.

The aim of this exploratory study is to generate hypothesis on and describe metastasis, the decisive cause of mortality that may be initiated during the interval between diagnosis and surgery for the female BC populations of the U.S. and Germany.

## Methods

The time from the confirmed diagnosis of an invasive BC until the start of treatment is referred to as the delay and includes the time required for organizational and technical processes. BC growth is an approximately continuous process, and to adequately estimate the effect of a delay of surgery, our model was built around a single biological parameter, the volume doubling time (VDT). The VDT depends, e.g., on grade or hormone receptor status (HR). VDT used by large study consortia are estimated via ultrasound, mammography, and reported for population-based mammography screening studies [6–9]. The association of HR and metastasis is also described by population-based data [10], and HR distributions and BC incidences are publicly available for several populations [11, 12].

The tumor diameter (TD) was furthermore considered as the central prognostic parameter, since the size of the TD at diagnosis describes the duration of the occult growth phase and thus the duration of the risk of distant metastasis. The TD is therefore considered a surrogate parameter which is correlated with metastasis and mortality. Each 5 mm interval of TD, for example, represents a subgroup of BCs with a specific distribution of prognostic factors and corresponding mortality. As the TD increases, the M1 findings increase, more positive lymph nodes are affected, and the proportion of grade G1 and HR + cases decrease. The association between TD and BC mortality can be described by population-based survival data for first single BC [12, 13].

The consequences of delay e.g., numbers of additional deaths are therefore estimated in dependence on HR-Status and delayed weeks based on these known parameters and using available aggregated population-based data. Therefore, power or sample size analysis, randomization, and blinding are not applicable.

### Data sources and assumption parameters

All calculations are based on female BC incidence data and distributions of delay from the U.S. (Delay data: study by Wiener et al., Incidence: NCI Cancer Report 2022) [3, 14] and Germany (Delay: report by the German Institute for Quality Assurance and Transparency in Health Care, Incidence: German Centre for Cancer Registry Data, 2019) [11, 15].

**Fig. 1:**
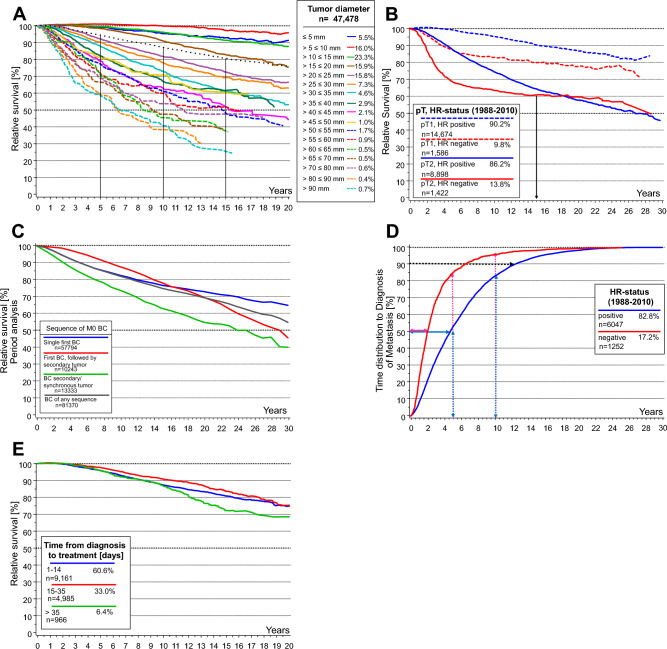
Long-term breast cancer survival based on data from the MCR, first single tumors only. **A:** Relative survival stratified by TD. The dotted line shows a relative survival for all M0 BCs of 80.7% at 15 years and 76% at 20 years (1998–2019, n = 47,478). **B:** Relative survival stratified by HR status (1988–2010, pT1 n = 16,260, pT2 n = 10,320). The faster growth of HR- MET causes different results in the pT1- and pT2-BC subgroups. **C:** Relative survival according to tumor sequence (Period analysis, BC M0, 1988–2018), only first single BCs adequately illustrate the BC-specific disease course, the other disease courses are impacted by preceding or secondary cancer diseases. **D:** Time to diagnosis of MET by HR status for first single M0 BC (diagnosed 1988–2010, n = 7,299). Within 25 years, all MET after primary M0 are diagnosed. 50% of MET in HR- are diagnosed within 2.1 years, in HR + within 5 years after initial diagnosis of the primary tumor. MET which are diagnosed soon after initial treatment are in a later stage of development and were initiated long before diagnosis (and treatment) of the primary BC, later diagnosed MET were initiated shortly before and were able to escape destruction by adjuvant systemic treatment. Therefore, twice the median time can be estimated as the median time of growth and development of MET, which are initiated during surgical delay until detection which is 10.8 years in HR + and 4.2 years in HR−. The growth time depends on tumor biology (above all HR status) and not on the size of the primary BC at the time of MET initiating tumor cell spread. Secondary BCs are excluded because MET from a contralateral BC, for example, would prolong the distribution of time to MET. **E:** Relative Survival of hormone receptor positive and M0 BC in dependence on days delayed (1998–2010, n = 15,112)

Relative survival as the best estimate for BC-specific survival in epidemiology, cumulative incidence of distant metastases (MET), and time distribution of metastasis by TD and HR were derived from the population-based Munich Cancer Registry (MCR) database (initial diagnosis 1988–2019, first single malignancies except in Fig. 1C). The MCR was the official regional cancer registry of Upper Bavaria from 1978 to 2021 and accounted for an area of 2.8 M up to 4.3 M since 2002, and 5.16 M inhabitants since 2007. By documenting all death certificates and actively following up the living, the MCR was able to estimate valid long-term population-based follow-up data on BC, which is rarely found elsewhere. Also, tumor-specific characteristics such as HR and TD were documented over decades. Since the German health care system offers broadly the same access everywhere, the MCR data are assumed to be representative for Germany.

Parameters and assumptions for modeling:

1.) Relative survival stratified by TD and HR is derived from MCR data, which includes follow-up data up to 20 years and more (see Fig. 1A, B). The association of 15-year BC-specific mortality (1 minus 15-year relative survival) and TD in M0 BC can be described by a Gompertz function (GF) [12].

$$GF:15 - yrMortality(\% ) = a*e^{{\left( { - b*e\left( { - c*TD} \right)} \right)}}$$

Here, a=58.4, describing an asymptote parallel to the x-axis which intersects the y-axis at 58.4; b=4.46, setting the displacement of the graph along the x-axis; and c=0.071 describing the growth rate.

For example, 15-year BC mortality increases by 18.1% with a tumor growth from 12 to 25 mm or by 1.39% per mm of growth with a turning point at 21.1 mm [13]. Increases in 15-year BC mortality between 8.4 and 26.6% follow a nearly linear association with tumor growth (see supplement Figure S1). This function describes for the most part a biological association which is derived from population-based tumor cases with any standard treatment approved during their period of diagnosis.

2.) The TD depends on the growth of the BC, and therefore on VDT for HR+ and HR−, respectively. HR− BCs grow faster than HR+ BCs, approximately by a factor of 2.4 (Fig.1D). The mean VDTs derived from two publications are 170/72 days for HR+ and HR− BCs [6, 7]. Considering the proportions of HR+/HR- (85%/15%) subtypes in the German and U.S. population, an approximated overall weighted mean VDT of 150 days was used for estimation. This VDT value is referred to in other studies as well [16].

Additional stratification by HER2+, Ki67, or neoadjuvant treatment modality was not conducted in this exploratory study. Ki67 was only implemented in widespread diagnostics in 2007. Nevertheless, limiting the modeling on TD and HR status broadly explains prognosis for a large majority of patients.

3.) The distribution of delay (in weeks) as it is available for the U.S. and Germany (see Table 2, Column 2 and 10) was implemented as influencing factor [3, 17]. Unknown frequencies for the first four weeks in the U.S. were split according to data from Germany. The middle of each week represents the duration of delay; 48/90 days were assumed for >6/ >12 weeks to model German and U.S. data.

4.) Female BC incidences for the U.S. and Germany in absolute numbers define the model cohorts [14, 15]. They are split into subgroups according to the distribution of clinical characteristics as primary M category, HR, and TD derived from MCR clinical data. Current data state that 6.2% of BCs are primary M1 and 85% are HR+. Exploratory analysis of SEER data shows similar proportions (5.8% M1 and 84.7% HR+) [11, 12].

5.)The distribution of the time to MET is determined by HR status. Based on MCR data, the 80% percentile intervals PI_80_(p_10_, p_50_, p_90_) of time to MET in M0 BC are PI_**80**_(16.9, 60.4, 141.3) months for HR+ and PI_80_(9.4, 25.2, 80.8) months for HR-(Fig. 1D) [10]. This implies that 50% of developing METs will be diagnosed within 5.0 or 2.1 years and 50% later than 5.0 or 2.1 years after initial diagnosis in M0/HR+ or M0/HR− BCs, respectively. This means that relevant, potentially preventable METs initiated during delay may be detected on average after at least twice the median MET-free time because growth from initiation to detectability may take about 10.0 / 4.2 years for HR+/HR− BCs.

The observable increase in mortality due to delay is lessened if the surgery is followed by an adjuvant therapy which eradicates a part of the MET initiated during the delay.

### Derivation of tumor growth

Based on the volume doubling of a spherical tumor with the initial diameter of 19.8 mm after 170/72 days at HR + and HR− the corresponding tumor diameter is calculated for weeks 1 to 8, as well as for 3 and 6 months of further growth (days 4, 11, 18, 25, 32, 39, 46, 53, see Table 1, rows 2 and 5 for HR + /HR− with different VDT of 170/72 days; for details see supplementary Figure S2):

$$TD_{d} = 19.8\times\,1.26^{{\left( {d/VDT} \right)}} \,\left( {with\,TD_{0} = 19.8\,mm\,and\,d = delay\,time\,in\,days} \right)$$

**Table 1:** Increase TD and 15-year mortality risk as a function of HR status and delayed surgery in weeks/days

Delay [weeks/ months/ days] Aspects: cohorts n = 1,000	Basis	1w 4 days	2w 11 days	3w 18 days	4w 25 days	5w 32 days	6w 39 days	7w 46 days	8w 53 days	3 m 90 days	6 m 180 days
HR +											
TD [mm]	19.80	19.91	20.10	20.29	20.48	20.68	20.88	21.08	21.28	22.32	25.26
15-yr mortality rate [%]	19.57	19.73	20.02	20.31	20.61	20.91	21.21	21.51	21.82	23.4	27.8
BC deaths per cohort [n / RR]	196	+ 2/ 1.008	+ 5/ 1.022	+ 7/ 1.038	+ 10/ 1.053	+ 13/ 1.068	+ 16/ 1.084	+ 19/ 1.100	+ 23/ 1.115	+ 38/ 1.19	+ 82/ 1.42
HR−											
TD [mm]	19.80	20.06	20.51	20.98	21.45	21.94	22.44	22.95	23.47	26.26	35.17
15-yr mortality rate [%]	19.57	19.96	20.65	21.36	22.09	22.83	23.59	24.36	25.15	29.26	40.46
BC deaths per cohort [n / RR]	196	44/ 1.020	+ 11/ 1.055	+ 18/ 1.091	+ 25/ 1.128	+ 33/ 1.167	+ 40/ 1.205	+ 48/ 1.245	+ 56/ 1.285	+ 97/ 1.49	+ 209/ 2.07

A TD of 19.8 presents the mean of the population-based all M0 findings in Fig. 1A. The mathematical derivation is described in Figure S1

*HR* hormone receptor status, *m* months, *TD* tumor diameter, *w* week(s), *yrs* years, *RR* relative risk

The cohorts of annual incident BCs (2022 U.S., 2019 Germany) with primary M0 (about 93.8% of the total cohort, because of elimination of 6.2% primary M1, n = 229,502 for the U.S. and n = 56,906 for Germany) are divided first into two theoretical cohorts by HR status, the HR + cohort comprises 85% of M0 and the HR- cohort 15%. These two cohorts are now split further into “delay cohorts” according to the current distribution of U.S. and German delay in weeks up to ≥ 12 weeks and ≥  7 weeks, respectively (see Table 2, Column 3 and 11). For each of these “delay cohorts,” the same mean TD of 19.8 mm is assumed at the time point of initial diagnosis with an average basal 15-year BC-specific mortality without delay derived from the Gompertz function shown above. The 15-year BC-specific mortality depending on the delay and estimated grown TD is also derived from this function for each “delay cohort.”

**Table 2:** Increase in TD as a function of HR + /HR- and delayed surgery in weeks and mortality caused by delay in the U.S. and Germany

Delay [weeks/days]		U.S.—Data 2022 Invasive BCs: M0 n = 270,002/ + M1 n = 17,850 ^a^						Delay [weeks/days]		German—Data 2019 Invasive BCs: M0 n = 66,949/ + M1 n = 4,425^b^			
		M0/HR +			M0/HR−					M0/HR +		M0/HR-	
	%	n^c^	Hazard Ratio (RR)	Deaths [n]	n	Hazard Ratio (RR)	Deaths [n]		%	n^d^	Deaths [n]	n	Deaths [n]
0	100	229,502	1.00	44,914*	40,500	1.000	7,926*	0	100	56,906	11,137*	10,043	1,965
1/4	2.0	4,589	1.008	+ 7	810	1.020	+ 3	1/4	2.5	1,445	+ 2#	255	+ 1#
2/11	11.2	25,635	1.023	+ 115	4,524	1.055	+ 49	2/11	14.2	8,075	+ 36	1,425	+ 15
3/18	17.8	40,864	1.038	+ 302	7,211	1.091	+ 129	3/18	22.6	12,872	+ 95	2,272	+ 41
4/25	15.8	36,203	1.053	+ 373	6,389	1.128	+ 160	4/25	20.0	11,404	+ 117	2,013	+ 51
5/32	15.4	35,376	1.068	+ 474	6,243	1.167	+ 204	5/32	14.8	8,439	+ 113	1,489	+ 49
6/39	11.6	26,709	1.084	+ 438	4,713	1.205	+ 189	6/39	9.8	5,577	+ 91	984	+ 40
7/46	8.2	18,899	1.100	+ 369	3,335	1.245	+ 160	> 7/46	16.0^e^	9,094	+ 186	1,605	+ 81
8/53	5.7	13,021	1.115	+ 293	2,298	1.285	+ 128	_^f^	_	_	_	_	_
9/60	3.8	8,751	1.131	+ 224	1,544	1.327	+ 99	_	_	_	_	_	_
10/67	2.5	5,783	1.147	+ 167	1,021	1.367	+ 73	_	_	_	_	_	_
11/74	1.6	3,782	1.164	+ 121	667	1.410	+ 53	_	_	_	_	_	_
12/81	1.1	2,562	1.179	+ 90	452	1.452	+ 40	_	_	_	_	_	_
> 12/90	3.2^e^	7,328	1.201	+ 288	1,293	1.507	+ 128	_	_	_	_	_	_
Median 33.7 days	100	229,502	1.073	+ 3,261*	40,500	1.179	+ 1,415*	Median 26 days	100	56,906	+ 640	10,043	+ 278

6.2% for primary advanced BCs (M1), 85% for HR + , and 19.8 mm TD are assumed. When calculating with a 20.8 mm TD, the values marked with * result higher: In the U.S. with HR + /HR− 48.402/8.541 deaths and 3.429/1.483 deaths by delay, in Germany 12.001/2118 deaths and 675/289 deaths by delay. Example: 40.864 M0/HR + BC with a TD of 19.8 mm have the basal 15-year BC-specific mortality (19.57%) =  > 7,997 deaths, with a delay of 18 days the tumor can grow up to a TD of 20.29 mm with a 15-year BC-specific mortality of 20.31% =  > 8,299 deaths, 8,299 minus 7,997 deaths under basal mortality =  > 302 additional deaths.

(a) BC incidence for the U.S. [11], (b) for Germany [15] Real world data of delays: (c) n = 373,334 from 2010 to 2014 [3], (d) n = 51,724 from 2020 to 21[17], (e) may not sum up to 100% due to rounding, (f) Longer delay grouped at > 7 weeks due to patient numbers. # the same RR as in the US cohort are also applied in the German cohort.

(b) *BC* Breast cancer, *HR* Hormone receptor status, *TD* tumor diameter, *VDT* Volume doubling time (72/170 days for HR−/HR +).

The difference in both mortalities reflects the additional delay effect, which is shown in Tab. 1, rows 4 and 7 as relative rate (RR: 15-year mortality after delay divided by the baseline 15-year mortality) and absolute excess deaths after 15 years. The 15-year BC-specific mortality in M0 BC is considered a suitable endpoint, because of the known course of BC disease from initial diagnosis of the primary tumor to the detection of further MET and the survival after MET. The duration of MET growth and the survival time from MET determine the time from which any delay-initiated MET occur or rather can be detected.

To determine the variability of the association between delay and underlying assumptions of VDT and TD, estimations were repeated for three different VDT (150–190, 62–82) and different basal TD, a mean TD of 14 mm as it is found in screening cohorts, and a mean TD of 28 mm found in a pT2-BC cohort (see supplementary Table S4).

This can be considered a deductive approach whereby a verifiable thesis is derived from public data sources.

### Time-to-event and other statistical analyses

All time-to-event analyses were completed with data from the MCR breast cancer population (1988–2018). For reference, the demographic data of this cohort are shown in supplementary table S3. Due to legal changes in cancer registry proceedings for Upper Bavaria, MCR high-quality follow-up data are only available until the 2018 patient cohort (censored on March 31, 2019). They were conducted as part of the established routine data preparation until end of 2021 according to public mandate.

Relative survival is calculated from observed survival estimated using the Kaplan–Meier method and the respective expected survival of the German general population using the official age- and sex-matched life tables (Ederer II method). Secondary malignancies, especially contralateral BCs, are excluded from analysis since the METs originating from these cases would falsify the estimates for the primary diagnosis (see Fig. 1C). Time to MET was estimated by cumulative incidence analysis considering competing risks (e.g., death) [18]. In addition, hazard ratios of the delay effect on overall survival are estimated using a multiple Cox proportion hazard model adjusting for age, TD, tumor grade, molecular subgroups, and number of positive lymph nodes (See supplementary Table S5). Survival analyses and Cox regression were performed in SAS V 9.4. For the estimation and delay calculations, the R system version 3.1.3 was used and results were validated in SAS [19]. The R code for the delay calculations can be found in the supplementary materials. For all analyses, a two-sided p-value of 0.05 or less was considered statistically significant. Plots were generated in SAS V 9.4.

## Results

In Germany, 51% of BC patients were treated within 14 days until 2006, this proportion decreased to 17% in 2017 indicating an increase in time between initial diagnosis and start of treatment as mentioned in the introduction. About 53.2% were treated after a delay of more than 4 weeks in the U.S. in 2022.

In 2020, the number of annual new cases were 287,850/71,375 BCs in the U.S. and Germany, respectively. The exclusion of 6.2% primary M1 cases results in 270,000/66,950 T-N-M0 BCs. These represent the at-risk populations, which could be affected by surgical treatment delay (Table 2). Table 1 describes tumor growth depending on delay in HR + and HR− BC with a TD of 19.8 mm at initial diagnosis and a VDT of 170/72 days for HR + /HR− BCs. For example, a HR + BC reaches a TD of 20.68 mm within five weeks or up to day 32. A HR− BC would have grown to a TD of 21.94 mm in the same time interval because of the faster VDT, assuming the same TD of 19.8 mm at diagnosis. Applying the Gompertz function for 15-year BC-specific mortality results in a mortality of 20.91%/22.83% for HR + /HR− BCs after a surgical treatment delay of five weeks. In relation to the base value for a 15-year mortality of 19.57% without delay this corresponds to an increase of 6.8%/16.7% (RR 1.068/1.167) for HR + /HR− BCs, respectively (Table 1). Considering 1,000 BCs treated without delay 196 BC-specific deaths are expected after 15 years, a delay of five weeks would result in an estimated 13/33 additional delay-associated deaths for HR + /HR− BCs. Extrapolated to the U.S. BC population an estimated additional 5,165/2,259 BC deaths would be associated with a delay of 8 weeks for all surgical procedures (see Table 1). The demographic and clinical characteristics of the underlying BC populations can be found here: SEER (https://seer.cancer.gov/statfacts/, https://seer.cancer.gov/statistics-network/explorer/), German BC population (https://www.krebsdaten.de/Krebs/EN/Content/Publications/Cancer_in_Germany/cancer_in_germany.html), MCR (supplementary Table S3).

The results are replicated in a multiple Cox model of OS with a continuous independent delay variable (delay in days 1.004 [95%CI 1.002–1.005], p < 0.0001) (supplementary Table S5). Negative HR status or larger TD were not associated with a shorter treatment waiting period (supplementary Table S3).

The delay stratified according to HR + /HR- can be associated with an estimated additional 3,261/1,415 deaths in the U.S. and 640/278 deaths in Germany. Based on these results, two conclusions can be drawn:

Firstly, after 15 years the prognosis worsens by 1.6%/1.2% for the U.S. and Germany, respectively. The difference results from the different distributions of treatment delay with a mean of 33.7 days in the U.S. and 26.0 days in Germany. These delay effects vary with the assumptions of VDT, 15-year relative survival, and TD. Changes in mortality based on different assumptions of VDT and TD at initial diagnosis can be seen in Table S3. The delay effect as a biological tumor-inherent effect is relevant in screening underlying populations as well. This is because confirming a finding with biopsies and referral for treatment can further delay primary treatment. If the mean TD in a screening population is about 14 mm vs. 19.8 mm, then a 15-year mortality of 11.2% vs. 19.57% is to be expected. With an 8-week delay, mortality would increase by 12%/31% for HR+/HR- BCs.

Secondly, reference to the at-risk populations HR + / HR- M0 BCs yields an increase of 7.3%/19.7% (RR 1.073/1.197) and 5.7%/14.1% in BC-associated deaths (U.S./Germany; see Table 2).

In Table 2, the population is the only varying variable. The other factors such as HR status, increase in TD with delay, association of TD with prognosis, and proportion of M1 remain unchanged. The mortality caused by the delay can be calculated from available data: Mortality without delay sums up to 70,690/17,527 and with delay to 75,366/18,445 for the U.S. and Germany. Relative to the absolute incidence numbers, this results in a 15-year BC-specific mortality of 26.2/25.8%. In Germany, 18,425 BC-dependent deaths were registered in 2020 [15]. In the U.S., only 43,250 BC deaths are estimated in 2022 [11].

The time distribution of the deaths caused by surgical treatment delay can be inferred from the distributions of MET-free times (Fig. 1D). Since these METs are initiated during the delay interval, their median growth time—about twice the MET-free times of 60.4/25.2 months mentioned above—and the 28 months of additional median survival from MET diagnosis onwards results in median survival times of about 12.4/6.5 years of delay-related deaths who contribute to mortality up to 30 years which is also suggested by the literature (Fig. 1B, E) [20, 21]. Only a few MET occur 20 years after diagnosis, but they may still be the cause of death after 25–30 years.

## Discussion

In this modeling study publicly available, real-world data are used to estimate and compare the risks associated with surgical treatment delay in both the U.S. and Germany (Table 2). Based on the currently available data on delay, the risk for subsequent metastasis and delay-associated BC-specific mortality was estimated based on tumor diameter, HR status, and treatment delay. The results of our analysis show that BC-specific survival decreases by an estimated 1.6/1.2% for the U.S. and Germany, respectively. Based on these estimates of the delay effect it can be assumed that every day counts until initial surgery. Depending on HR status postponing surgical treatment by the proposed safe interval of eight weeks as suggested by Wiener et. al. would result in an additional 5,170 HR + /2,260 HR- BC deaths in the U.S. These findings are in line with the previous research, which revealed an increasing time interval between the first physician visit and tumor removal [2, 5]. A subsequent assessment of the outcome from 2016 found an increase in all-cause mortality of 9% for each month of delay [22, 23].

The results are based on a comprehensible transformation of valid data and applies to both the U.S. and Germany.

The results are stratified according to TD and HR status and can be estimated for any country where data for these factors are available. They define subgroups which describe the TD-dependent metastasis in the occult phase. Survival is therefore a characteristic parameter for each TD. The expected additional mortality with the delay can then be estimated using epidemiological and publicly available data for several health care systems. However, the frequency of the respective subgroup depends on the population, e.g., on the use of screening. Therefore, if there are differences in the frequency of the subgroups of T-N-M0 BCs, the mean TD must be adjusted for the calculation of the delay effect. As for the U.S. and Germany, in Table 2, a TD of 19.8 mm was assumed for the comparison because the proportion of M1 at 6.2% and HR + status at 85% remain the same over time in both countries. Similarly, RS according to TD is nearly identical in both countries up to 10 yrs after diagnosis.

The same may be said regarding current therapy options which should result in similar survival. Long-term relative survival as a function of HR status is similar after 15 years (see Fig. 1B for estimates for the German population) [10, 24]. This is also shown by the distributions of MET-free times and the ratio of their medians 60.4/25.2 or 2.40 (see Fig. 1D for estimates for the German population) as the ratio 2.36 of VDTs adopted for HR + /HR- of 170/72. The median TD of 19.8 mm used in our study for M0 BCs remains constant and transferable because the pT-category distribution is also comparable in both populations [12].

Considering VDT and the functional relationship between TD and survival, the effects of early and delayed detection of BC can be quantified. Successful early detection leads to saved lives by ending further tumor growth and metastasis and vice versa every delayed treatment and further tumor growth lead to lives lost. This balance between lives gained and lost can be calculated. Within two weeks, the TD of a HR + M0 BC of 19.8 mm may grow by 0.38 mm and the mortality risk increases mathematically by 0.0058%. However, this risk, which is not relevant in the individual case, would already implicate 1,328 additional BC deaths after 15 years out of 229,502 HR + /M0 BCs, which are diagnosed annually in the U.S. (Table 2). This exemplifies that a very small risk in a large population, 2.3 M new BC patients per year worldwide, can be more severe than a large risk in a small population.

The continuous MET risk can also be derived from a further consideration: if a BC does not initiate MET for eight weeks after detection, then this would also be the case if the BC was not detected. The MET risk cannot depend on detection itself, provided there is no neoadjuvant systemic therapy [3]. If BC was diagnosed right after this time point, there would be another eight weeks and any number of recurrent weeks without risk of MET. However, a risk-free interval and the process of MET are mutually exclusive. MET is a continuous process which follows the principle of “natura non facit saltus.”

If there is no risk-free delay, a significant delay limit at which undertreatment begins cannot be specified. The time interval between diagnosis and initial surgical treatment determines the potential MET initiation and therefore a prolonged interval implicates an excess mortality after a median of 6.5/12.4 years (sum of median time to MET plus 2.2 yrs of median survival afterward). In general, the risk associated with a delay in surgical treatment and the underlying MET process can only be quantified considering up to 30 years of BC survivorship surveillance. Five to ten years of follow-up are not sufficient in determining the delay-associated risk. With a five-year follow-up and 373,334 patients, this association is unlikely to be proven even for triple-negative BC [3]. The last occurring MET in single BCs—without consideration of contralateral BCs—can be expected up to 25 years (Fig. 1D), and therefore, BC mortality should be assessed considering a 30-year follow-up.

The shortest possible time to treatment should also be considered in tumors diagnosed by mammography screening. This is because delay to surgery starts with the initial suspicious finding and its potential tumor cell dissemination. Along the estimated trajectories, each time span adds up until the definite histological confirmation and subsequent treatment initiation.

Our analyses and conclusions carry inherent limitations. One lies in the dependence of the delay effect on the basic distribution of M1, HR status, TD, and the VDT. Especially TD may vary in populations due to screening programs and influences the mean TD of a population. Although the survival in a TD subgroup would remain the same, survival, however, depends on delay and VDT, for which we calculated deviations of 15% of the HR ± VDT of 72/170 days. Because of innovations in treatment and diagnosis, the cure fraction, which can be estimated by the complement of the relation of BC mortality and incidence, varies. The official mortality of a year approximately summarizes disease courses which began a few months or up to 30 years ago. Here, the period survival can be used to project disease courses of a year into the future from a few months up to 30. The calculations for the delay cohorts of Table 2 can be demonstrated with historical cohorts (Fig. 1D) but cannot be confirmed experimentally with a randomized trial. However, this does not call into question the dependence on TD and VDT, and subsequently, the downstream delay effect. Further important prognostic factors such as histological subtypes with consideration of HER2-receptor status, Ki67, and grade of differentiation were not considered in this study. However, HR status as a prominent factor that represents the different distributions of these prognostic factors was included in the estimations by considering different VDT. It should be noted that a reasonably prioritized treatment of delay-sensitive HR- and larger BCs is not evident in our data (Supplement Table S3).

Interestingly, our results for the U.S. estimate 70,687 BC deaths per year without delay (including M1) and a mortality of 24.6% after 15 years which would appear plausible. However, this is inconsistent with the officially reported 13.6% or 42,250 BC-specific deaths [11, 14, 25]. The SEER-reported 10-year relative survival of 85.4% should result in an estimated 45,365 deaths [26]. With our modeled rate of 86.5%, the 42,250 deaths would have already been reached after 8.9 years [27].

The delay time can be important both for the health care system and the patient. On the one hand there are possible sociodemographic and socioeconomic factors, preoperative diagnostic procedures such as imaging, histopathological examination, and a comprehensive examination of the patient to determine which treatment options can be offered, their prospects of success, and an assessment of the patient’s physical and psychological condition [2]. From the patient's point of view, this time is important to come to terms with the diagnosis and treatments, perhaps with a second opinion, and to mobilize coping strategies [28].

However, in each individual case, unnecessary delays should be avoided without exerting special pressure concerning the risk of mortality. In a modeling study such as this, the results offer estimates and approximations to be used as a basis to develop hypotheses on improvement of treatment pipelines, which would need to be tested with prospective analyses on ease of clinical implementation and effectiveness. For instance, if prolonged times between first diagnosis and surgery do not serve to improve treatment overall but emerge as bottlenecks, prioritization by HR and TD should be considered and undertaken. Alternatively, neoadjuvant endocrine therapies in particular could be administered [29]. Since the treatment sequences a) neoadjuvant chemotherapy → surgery and b) surgery → adjuvant chemotherapy are equivalent in regard to effect on MET initiation, the consequences of necessary delays can be reduced by prioritizing a neoadjuvant approach. It would reduce time pressure for scheduling surgery and present an advantage with reference to strength of response and TD reduction.

It can be concluded that there is no risk-free time from diagnosis to treatment. A growing tumor can continuously initiate MET, and therefore poses a relevant risk impacting prognosis with every additional millimeter. Confirmed BCs treated according to HR status as soon as possible could improve long-term prognosis by merely addressing the factor time.

## Supplementary Information

Below is the link to the electronic supplementary material.

Supplementary file1 (DOC 800 KB)

## Data Availability

The datasets used to support the results of this study are publicly available: SEER: https://seer.cancer.gov/statistics-network/ RKI: https://www.krebsdaten.de/Krebs/DE/Datenbankabfrage/datenbankabfrage_stufe1_node.html MCR: Munich Cancer Registry http://www.tumorregister-muenchen.de/en/facts/specific_analysis.php Publications: see reference section.
