# Supplementary material for: Prolonged time to breast cancer surgery and the risk of metastasis: an explorative simulation analysis using epidemiological data from Germany and the USA
Source: Breast Cancer Res Treat. 2025 Feb 17;211(1):151–60. doi: 10.1007/s10549-025-07630-9 (PMC11953083; doi:10.1007/s10549-025-07630-9)
Supplement: Supplementary file 1 — Supplementary file1 (DOC 800 KB) [file 10549_2025_7630_MOESM1_ESM.doc]

Supplementary Appendix for Breast Cancer Research and Treatment to

**Prolonged time to breast surgery increases risk of metastasis. An explorative simulation analysis using epidemiological data from Germany and the USA**

Dieter Hölzel^1*^, Anne Schlesinger-Raab^1*^, Gabriele Schubert-Fritschle^1^, Kathrin Halfter^1^

Corresponding author:

Dr. Kathrin Halfter (ORCID 0000-0002-5974-8959)

Institute of Medical Information Processing, Biometry and Epidemiology (IBE), Faculty of Medicine, LMU Munich, Marchioninistraße 15, Munich, 81377, Germany.

Email: halfter@ibe.med.uni-muenchen.de Telephone: 089 4400 74486

Table of Contents

**Figure S1.** Modeling the relationship between tumor size and 15-year BC-specific mortality. page 2

**Figure S2.** Modelling of tumor growth and variability depending on initial tumor size and volume doubling time. page 3

**Table S3.** Cohort of first single primary M0 invasive breast cancer 1998-2010 stratified by delay between initial diagnosis and treatment (MCR data). page 4-5

**Table S4.** Estimated tumor growth and 15-year excess mortality according to different tumor diameters at initial diagnosis and volume doubling times showing the variability (simplified program code in R attached) page 6-8

**Table S5.** Cox proportional hazard regression model (multivariable) including delay in days as a continuous variable, adjusting for age, tumor diameter, grade, hormone receptor and HER2 status and lymph node involvement in primary M0 BC page 9

**Figure S6.** Variability results: estimated 20-Year mortality and excess mortality depending on delay, hormone receptor status, and different initial tumor size, data according to Tab. S4. page 10

**Figure S7.** Variation in mortality for T-N-M0 BCs caused by delay as a function of varying VDTs for TD of 19-21mm and based on the estimated incident cases in the US and Germany. page 11

**Table S8.** Data sources and model assumptions. page 12

**Figure S1.** Modeling the relationship between tumor size and 15-year breast cancer specific mortality. Data as in Fig. 1A: data points are taken from the middle of each interval of tumor diameter and 15-year relative survival as 15-year BC specific mortality (1 minus 15-year relative survival, e.g. (3 mm | 7.1%) with a 15-year relative survival of 92.9% for tumors of a diameter of >0-5 mm (blue diamonds), the light blue and grey points correspond to the lower (LL) and upper limits (UL) of the 95% confidence interval of relative survival as ‘1 minus LL’ and ‘1 minus UL’). The red graph corresponds to the fitted Gompertz function.

**Figure S2.** Modelling of tumor growth and variability depending on initial tumor size and volume doubling time. The tumor growth calculations assume the tumor to be an ideal sphere (simplified):

Formulas A and B describe the relation between TD and Volume. The volume grows exponentially. Formulas C and D describe the relation between grown TD and delay with an initial TD of 19.8 mm. y corresponds to TD in mm, x to delay in days divided by VDT.

Vice versa, the duration of growth from one cancer cell with a TD of 10 µm (0.01 mm) to a tumor with 15 mm TD at initial diagnosis can be calculated as follows:


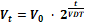
 ⇔ describes the growth factor after a defined time t


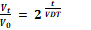
 ⇔ Equivalent transformation


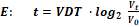
Resolution to t


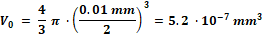


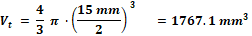


V_t_ and V_0_ are inserted in **E** under a condition of a VDT of 150 days, the result is t = 4,749 days, which corresponds to 13 years.

**Table S3:** Cohort of first single primary M0 invasive breast cancer, year of diagnosis1998-2010 and stratified by delay between initial diagnosis and treatment (MCR data). 4.6% of patients with more than 35 days of delay are not representative of current delay data, see table 2 for current delay data: US 37.8% with delay of > 35 days in 2022, Germany 25.8% with delay > 35 days in 2019.

| **Time of delay in days** | **n.a. / 0 days** | | **1-14 days** | | **15-35 days** | | **> 35 days** | | **Total** | | **p-value** |
| --- | --- | --- | --- | --- | --- | --- | --- | --- | --- | --- | --- |
|  | **n=7062 (28.9%)** | | **n=10562 (43.2%)** | | **n=5693 (23.3%)** | | **n=1135 (4.6%)** | | **n=24452 (100%)** | |  |
| **Prognostic factors** | **n** | **(%) ^a^** | **n** | **(%) ^a^** | **n** | **(%) ^a^** | **n** | **(%) ^a^** | **n** | **(%) ^a^** |  |
| **Age** mean ± SD | 61.5 | ± 13.7 | 61.2 | ± 13.2 | 60.8 | ± 12.5 | 61.7 | ± 14.2 | 61.2 | ± 13.2 | 0.0798 n.s. |
|  |  |  |  |  |  |  |  |  |  |  |  |
| **Age in classes** < 50 yrs. | 1532 | (21.7) | 2368 | (22.4) | 1217 | (21.4) | 254 | (22.4) | 5371 | (22.0) | <0.0001 |
| 50-59 yrs. | 1711 | (24.2) | 2415 | (22.9) | 1379 | (24.2) | 253 | (22.3) | 5758 | (23.6) |  |
| 60-69 yrs. | 1913 | (27.1) | 3056 | (28.9) | 1843 | (32.4) | 330 | (29.1) | 7142 | (29.2) |  |
| 70-79 yrs. | 1197 | (17.0) | 1826 | (17.3) | 890 | (15.6) | 168 | (14.8) | 4081 | (16.7) |  |
| ≥ 80 yrs. | 709 | (10.0) | 897 | (8.5) | 364 | (6.4) | 130 | (11.5) | 2100 | (8.6) |  |
|  |  |  |  |  |  |  |  |  |  |  |  |
| **c/p T-Category** T1 | 4230 | (59.9) | 5984 | (56.7) | 3420 | (60.1) | 612 | (53.9) | 14246 | (58.3) | <0.0001 |
| T2 | 2180 | (30.9) | 3771 | (35.7) | 1782 | (31.3) | 371 | (32.7) | 8104 | (33.1) |  |
| T3 | 253 | (3.6) | 401 | (3.8) | 255 | (4.5) | 73 | (6.4) | 982 | (4.0) |  |
| T4 | 267 | (3.8) | 363 | (3.4) | 177 | (3.1) | 62 | (5.5) | 869 | (3.6) |  |
| n.a. | 132 | (1.9) | 43 | (0.4) | 59 | (1.0) | 17 | (1.5) | 251 | (1.0) |  |
|  |  |  |  |  |  |  |  |  |  |  |  |
| **c/p N-Category** N0 | 4137 | (58.6) | 6399 | (60.6) | 3657 | (64.2) | 673 | (59.3) | 14866 | (60.8) | <0.0001 |
| N+ | 2256 | (32.0) | 3789 | (35.9) | 1897 | (33.3) | 390 | (34.4) | 8332 | (34.1) |  |
| NX | 527 | (7.5) | 355 | (3.4) | 130 | (2.3) | 68 | (6.0) | 1080 | (4.4) |  |
| n.a. | 142 | (2.0) | 19 | (0.2) | 9 | (0.2) | 4 | (0.4) | 174 | (0.7) |  |
|  |  |  |  |  |  |  |  |  |  |  |  |
| **Grade** G1 | 954 | (13.5) | 1305 | (12.4) | 822 | (14.4) | 160 | (14.1) | 3241 | (13.3) | <0.0001 |
| G2 | 3876 | (54.9) | 6099 | (57.7) | 3388 | (59.5) | 655 | (57.7) | 14018 | (57.3) |  |
| G3 | 2062 | (29.2) | 3049 | (28.9) | 1442 | (25.3) | 310 | (27.3) | 6863 | (28.1) |  |
| n.a. | 170 | (2.4) | 109 | (1.0) | 41 | (0.7) | 10 | (0.9) | 330 | (1.4) |  |
| SD: standard deviation, n.s.: not significant as defined by a level α set at 0.05, n.a. not available  **^a^** Missing values were not excluded from calculations of frequency distribution, column percentage can differ slightly from 100% due to rounding. | | | | | | | | | | | |

continuing

Tables S3. continued

| **Time of delay in days** | **n.a. / 0 days** | | **1-14 days** | | **15-35 days** | | **> 35 days** | | **Total** | | **p-value** |
| --- | --- | --- | --- | --- | --- | --- | --- | --- | --- | --- | --- |
|  | **n=7062 (28.9%)** | | **n=10562 (43.2%)** | | **n=5693 (23.3%)** | | **n=1135 (4.6%)** | | **n=24452 (100%)** | |  |
| **Prognostic factors** | **n** | **(%) ^a^** | **n** | **(%) ^a^** | **n** | **(%) ^a^** | **n** | **(%) ^a^** | **n** | **(%) ^a^** |  |
| **Hormone** HR+ | 5747 | (81.4) | 9161 | (86.7) | 4985 | (87.6) | 966 | (85.1) | 20859 | (85.3) | <0.0001 |
| **receptor** HR- | 778 | (11.0) | 1236 | (11.7) | 635 | (11.2) | 145 | (12.8) | 2794 | (11.4) |  |
| **status** n.a. | 537 | (7.6) | 165 | (1.6) | 73 | (1.3) | 24 | (2.1) | 799 | (3.3) |  |
|  |  |  |  |  |  |  |  |  |  |  |  |
| **HER2** HER2+ | 645 | (9.1) | 1205 | (11.4) | 684 | (12.0) | 150 | (13.2) | 2684 | (11.0) | <0.0001 |
| **status** HER2- | 3433 | (48.6) | 7399 | (70.1) | 4427 | (77.8) | 823 | (72.5) | 16082 | (65.8) |  |
| HER2 unclear | 234 | (3.3) | 363 | (3.4) | 139 | (2.4) | 32 | (2.8) | 768 | (3.1) |  |
| n.a. (mostly before 2004) | 2750 | (38.9) | 1595 | (15.1) | 443 | (7.8) | 130 | (11.5) | 4918 | (20.1) |  |
|  |  |  |  |  |  |  |  |  |  |  |  |
| **Subtype** Luminal-A like | 1463 | (20.7) | 4567 | (43.2) | 3054 | (53.6) | 547 | (48.2) | 9631 | (39.4) | <0.0001 |
| Luminal-B like HER2- | 363 | (5.1) | 1241 | (11.8) | 793 | (13.9) | 139 | (12.3) | 2536 | (10.4) |  |
| Lumnal-B like HER2+ | 253 | (3.6) | 725 | (6.9) | 444 | (7.8) | 90 | (7.9) | 1512 | (6.2) |  |
| HER2- non-luminal | 94 | (1.3) | 261 | (2.5) | 170 | (3.0) | 40 | (3.5) | 565 | (2.3) |  |
| Triple negative | 202 | (2.9) | 600 | (5.7) | 352 | (6.2) | 79 | (7.0) | 1233 | (5.0) |  |
| n.a. (mostly before 2004) | 4687 | (66.4) | 3168 | (30.0) | 880 | (15.5) | 240 | (21.2) | 8975 | (36.7) |  |
|  |  |  |  |  |  |  |  |  |  |  |  |
| **Surgery** Breast conserving | 4929 | (69.8) | 8095 | (76.6) | 4259 | (74.8) | 765 | (67.4) | 18048 | (73.8) | <0.0001 |
| Mastectomy | 1747 | (24.7) | 2394 | (22.7) | 1402 | (24.6) | 359 | (31.6) | 5902 | (24.1) |  |
| n.a. | 386 | (5.5) | 73 | (0.7) | 32 | (0.6) | 11 | (1.0) | 502 | (2.0) |  |
|  |  |  |  |  |  |  |  |  |  |  |  |
| **Neoadjuvant** yes | 343 | (4.9) | 278 | (2.6) | 419 | (7.4) | 139 | (12.3) | 1179 | (4.8) | <0.0001 |
| systemic treatment no | 6719 | (95.1) | 10284 | (97.4) | 5274 | (92.6) | 996 | (87.8) | 23273 | (95.2) |  |
| SD: standard deviation, n.s.: not significant as defined by a level α set at 0.05, n.a. not available  **^a^** Missing values were not excluded from calculations of frequency distribution, column percentage can differ slightly from 100% due to rounding. | | | | | | | | | | | |

**Table S4.** Estimated tumor growth and 15-year excess BC-specific mortality according to different tumor diameters at initial diagnosis and volume doubling times showing the variability (simplified program code in R attached)

Attached program code for R, see table S4.

**# Estimation of Delay**

mvd<- matrix(c(150,62, 170,72,190,82),nrow=3,ncol=2,byrow=T) # VDT for 3 HR-Status

a<-58.4; b<- -4.46 ; c<- -0.071 # Gompertz function

metp <- 0.062 ; # Proportion M1

hrg<- c(0.85,0.15) # Proportion HR %-pos/ %-neg

del <- c(0,4,11,18,25,32,39,46,53,60,67,74,81,88) ; ws<- length(del) # delay in days for 13 weeks

# Matrix: ff, TD, mvd, TD/15 J Surv, 13 weeks

vtd <- c(seq(5, 19,1), 19.8, seq(20, 30,1)) ;ztd<- length(vtd) # for tumor diameter from 5 to 28

res<- matrix(NA, nrow=12*ztd, ncol=ws+3)

z<-1

for ( k in vtd) { # TD 5mm – 28 mm

for (m in 1:3) { # VDT HR+ / HR-

for (n in 1:2) { # growth TD/week increase mortality/week

res[z,1] <- z ; res[z,2] <- k ; res[z,3] <- mvd[m,n]

tdd <- round(k*1.26^(del/mvd[m,n]),2) ; res[z,4: (4+ws-1)] <- tdd

z<-z+1

res[z,1] <- z ; res[z,2:3] <- res[(z-1),2:3] ; res[z,4: (4+ws-1)] <- round(a*exp(b*exp(c*tdd)) ,2)

s15y <- round(a*exp(b*exp(c*k)) ,2) ; res[z,4:(4+ws-1)] <- res[z,4:(4+ws-1)] - s15y; res[z,4]<-s15y

z <- z+1

}}}

**Table S5.** Cox proportional hazard regression model (multivariable) — overall survival and delay in days as a continuous variable, adjusting for dummy-coded variables age, TD, grade, HR and HER2 status and lymph node involvement in primary M0 BC (n=26,574/ events=4,144, 2007-2019, only cases with known HR-status and HER2-Status)

| **Factor** | **Hazard Ratio** | **95% Confidence**  **Interval** | | | **Chi^2^** | **p-value*** |
| --- | --- | --- | --- | --- | --- | --- |
| **Delay (days)** | **1.004** | **[1.002** | **- 1.005]** | **15.66** | | **<.0001** |
| Age (years) < 40 | Ref. |  |  | 1812.79 | | <.0001 |
| 40-49 | 0.735 | [0.603 | - 0.896] |  | |  |
| 50-59 | 0.885 | [0.732 | - 1.068] |  | |  |
| **60-69** | **1.327** | **[1.107** | **- 1.590]** |  | |  |
| **70-79** | **2.477** | **[2.073** | **- 2.961]** |  | |  |
| **≥ 80** | **5.975** | **[4.974** | **- 7.177]** |  | |  |
| TD (mm) ≤ 5 | Ref. |  |  | 383.23 | | <.0001 |
| 6-10 | 0.737 | [0.588 | - 0.924] |  | |  |
| 11-15 | 0.974 | [0.790 | - 1.201] |  | |  |
| 16-20 | 1.220 | [0.989 | - 1.505] |  | |  |
| **21-25** | **1.472** | **[1.196** | **- 1.813]** |  | |  |
| **26-30** | **1.530** | **[1.229** | **- 1.905]** |  | |  |
| **31-35** | **1.722** | **[1.375** | **- 2.156]** |  | |  |
| **36-40** | **2.043** | **[1.608** | **- 2.595]** |  | |  |
| **41-45** | **2.213** | **[1.733** | **- 2.825]** |  | |  |
| **46-50** | **1.853** | **[1.383** | **- 2.483]** |  | |  |
| **51-55** | **2.090** | **[1.615** | **- 2.704]** |  | |  |
| **56-60** | **2.270** | **[1.691** | **- 3.048]** |  | |  |
| **61-65** | **2.368** | **[1.689** | **- 3.319]** |  | |  |
| **66-70** | **2.756** | **[1.969** | **- 3.859]** |  | |  |
| **71-80** | **1.971** | **[1.414** | **- 2.749]** |  | |  |
| **81-90** | **3.284** | **[2.339** | **- 4.609]** |  | |  |
| **> 90** | **3.634** | **[2.670** | **- 4.946]** |  | |  |
| **Tumor grade** 1 | Ref. |  |  | 76.32 | | <.0001 |
| **2** | **1.269** | **[1.115** | **- 1.444]** |  | |  |
| **3-4** | **1.690** | **[1.468** | **- 1.946]** |  | |  |
| HR + / HER2 - | Ref. |  |  | 169.27 | | <.0001 |
| HR + / HER2 + | 1.013 | [0.906 | - 1.133] |  | |  |
| HR - / HER2 + | 1.156 | [0.984 | - 1.359] |  | |  |
| **Triple negative** | **1.922** | **[1.739** | **- 2.124]** |  | |  |
| **pN0** | Ref. |  |  | 543.10 | | <.0001 |
| **pN+ 1-3 lymph nodes** | **1.320** | **[1.220** | **- 1.428]** |  | |  |
| **pN+ 4-9 lymph nodes** | **2.009** | **[1.815** | **- 2.225]** |  | |  |
| **pN+ > 9 lymph nodes** | **3.071** | **[2.752** | **- 3.427]** |  | |  |
| **pNx** | **2.475** | **[2.132** | **- 2.872]** |  | |  |
| **pN n.a.** | **2.137** | **[1.400** | **- 3.262]** |  | |  |
| * Wald-test (delay), linear hypotheses Wald-test in dummy-coded variables  TD tumor diameter HR hormone receptor status pN pathological involvement of lymph nodes | | | | | | |

**Figure S6.** Variability results: estimated 15-Year mortality and excess mortality depending on delay, hormone receptor status, and different initial tumor size, data according to Tab. S4. Black graphs are assigned to the HR+ cohort, red to the HR- cohort. **A** 20 mm as initial tumor size represents the median tumor size of the M0 BC cohort, 14 mm represents the median tumor size of a BC cohort diagnosed by screening mammography, 8 mm is the median tumor size in pT1b BCs. **B** The black graphs show the lower excess mortality in HR+ BC with longer volume doubling times and slower growth, red graphs correspond to HR- BC with shorter doubling times and faster growth, green graphs describe the weighted average (WA) excess mortality in an assumed fixed distribution of hormone receptor status of 85% HR+ and 15% HR-.

**Figure S7.**

Variability of mortality for T-N-M0 BCs caused by delay as a function of varying VDTs for TD of 19.8mm and based on the estimated incident cases in the US and Germany. The association of mortality and TD is just changed by the VDT resulting in a shift along the y-axis and leading to a smaller number of additional deaths with a longer VDT of 82/190 days (blue curve). A shorter VDT of 62/150 days would lead to a greater number of additional deaths (red curve). These curves could serve as a confidence interval for the estimation.

The expected deaths without delay as a function of TD between 16 and 24 mm are summarized for the U.S. and Germany. They form the basis for further delay-related mortality, whose dependence on the VDT is described by the curves. The TD seems plausible as 20 mm representing the median TD of the entire BC cohort. Provided that the prolonged delays occurred mainly in the last decade, most delay-related deaths will probably occur at a later time point and weaken the preventive and therapeutic successes in the future. The total deaths of a BC population result from the subpopulations M1, T‑N‑M0, and the additional delay-related deaths. The numbers of estimated deaths are consistent with official German figures (providing a mean TD of 20 mm and the prevalence of 50% of all delay-related deaths currently). U.S. data shows serious inconsistencies in various publications and well-founded estimates. ^1-3^ However, the expected deaths may increase by approx. 5%.

Table S8 Data sources and model assumptions

|  | Data Source | Measure/ derived measure | Assumptions |
| --- | --- | --- | --- |
| 1. | Munich Cancer Registry MCR  (population-based in Upper Bavaria)  **First single BC diagnosed 1998-2019**  Censored at 31.12.2021, n=47,478 with information on tumor diameter | Relative survival (RS) stratified by classified tumor diameter (TD) (Figure 1A)  **🡪** 1-15-year RS = 15-year BC specific mortality  **🡪** Gompertz function is fitted on tumor diameter and 15-year BC specific mortality (Figure S1)  GF: 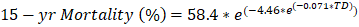 | - The biological association between TD and 15-year BC specific mortality can be described by a Gompertz function, which is used to assign the BC-specific 15-year mortality to every TD (by mm) - Tumor size is a measure of tumor growth duration - Tumor growth duration is associated with spreading of MET (and MET growth)   Cave: other prognostic factors are ignored for simplification |
| 2. | Ryu et al. 2014 ^4^, Weedon-Fejaer et al. 2008 ^5^, Chlebowski et.al. 2009 ^6^ | Tumor volume doubling times (VDT) from imaging studies  72 days in HR negative BC  170 days in HR positive BC  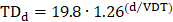  (with TD_0_ = 19.8 mm and d = delay time in days) | - The volume of BCs can be described as a spherical volume. TD and spherical volume and volume doubling time can be used to calculate the TD after a certain time of delay and further growth. - With different VDT and different median TD at initial diagnosis the TD after any delay can be calculated   🡪 a greater TD leads to greater 15-year BC specific mortality  Cave: VDT of molecular subgroups are not known |
| 3. | Distribution of delay (in weeks)  U.S. Wiener et al. 2023 ^7^  Germany IQTIG 2022 ^8^ | Time between initial diagnosis (histologic confirmation) and surgery in weeks | The distributions are representative for the U.S. and Germany |
| 4.a | U.S. SEER: Siegel et al. 2022 ^1^  Germany RKI: ZfKD 2019 ^2^ | Breast cancer statistics: estimated absolute numbers of female BC for 2022 in the U.S. and 2019 in Germany  The absolute numbers are distributed based on delay distribution for the U.S. and Germany each. | The published distributions of delay (3.) are representative for the U.S: and Germany. |
| 4.b | Munich Cancer Registry MCR as in 1. | Distributions of primary M-category and hormone receptor status.  M1: 6.2% and HR+ 85%  6.2% are subtracted from the data of 4.a and all subgroups by delay are divided into 85% HR+ and 15% HR-. | - Absolute numbers in the subgroups by delay are all divided into 85% HR+ and 15% HR- which introduced a simplification in the model. - This seems appropriate because there was no evidence for prioritization of certain subgroups to a shorter delay. |
| 5. | Munich Cancer Registry MCR  (population-based in Upper Bavaria)  **First single M0 BC diagnosed 1988-2019 who developed MET within 30 years**  Censored at 31.12.2021, n=7,299 | Time distribution of MET in the course of disease over 30 years in primary M0 BC stratified by hormone receptor status.  This shows that 50% of MET in HR+ BC are diagnosed within 5 years, further 30% within >5 to 10 years after initial diagnosis, further 20% later  In HR- 50% MET are diagnosed within 2.1 years, further 35% within >2 to 5 years, further 10% within >5 to 10 years and the last 5% later. | - MET precede death and are the cause of BC specific death. - The harmful thing about delay is the possibility for the primary tumor to continue to grow and spread MET. These MET initiated in the delay interval should occur in a later period of MET distribution according to their growth behaviour. - We postulate that these MET may be detected on average after at least twice the median MET-free time because the growth of lately initiated MET may take 10 years (2x5) in HR+ and 4.2 years (2x2.1) in HR-. - Long-term observation is at least required to prove the safety of delay in HR+ BCs. - The biological implausibility remains. |
